# Influences of Plant Traits on Immune Responses of Specialist and Generalist Herbivores

**DOI:** 10.3390/insects3020573

**Published:** 2012-06-19

**Authors:** Evan Lampert

**Affiliations:** Department of Biological Sciences, Gainesville State College, Oakwood, GA 30566, USA; E-Mail: elampert@gsc.edu; Tel.: +1-678-717-3804; Fax: +1-678-717-3770

**Keywords:** antioxidant, ecological immunity, encapsulation, Lepidoptera, secondary compounds, sequestration, tradeoff

## Abstract

Specialist and generalist insect herbivore species often differ in how they respond to host plant traits, particularly defensive traits, and these responses can include weakened or strengthened immune responses to pathogens and parasites. Accurate methods to measure immune response in the presence and absence of pathogens and parasites are necessary to determine whether susceptibility to these natural enemies is reduced or increased by host plant traits. Plant chemical traits are particularly important in that host plant metabolites may function as antioxidants beneficial to the immune response, or interfere with the immune response of both specialist and generalist herbivores. Specialist herbivores that are adapted to process and sometimes accumulate specific plant compounds may experience high metabolic demands that may decrease immune response, whereas the metabolic demands of generalist species differ due to more broad-substrate enzyme systems. However, the direct deleterious effects of plant compounds on generalist herbivores may weaken their immune responses. Further research in this area is important given that the ecological relevance of plant traits to herbivore immune responses is equally important in natural systems and agroecosystems, due to potential incompatibility of some host plant species and cultivars with biological control agents of herbivorous pests.

## 1. Introduction

Interest in questions related to ecological immunology arose relatively recently as a synthesis between evolutionary ecology and immunology, and has grown dramatically in the last decade [1]. Ecological immunology refers to the study of how a combination of biotic and abiotic factors influences the immune responses of organisms [1,2]. Much research in ecological immunity has focused on tradeoffs between these biotic and abiotic factors and immune response [3]. Especially well-studied ecological factors that can compete with immune response include limited resource availability (poor nutritional status, *sensu *[4]) and competing physiological needs (such as reproductive status, *sensu* [5]) of the target organism.

Rolff and Siva-Jothy [6] discussed the need for more research in ecological immunity of invertebrates, in large part because evolutionary ecology of the immune response is easiest researched in organisms with simpler immune responses. Insects, like other invertebrates, rely primarily on innate immune responses to survive attacks by pathogens and endoparasites [7,8]. Insect models such as *Drosophila melanogaster *and *Manduca sexta *have been used extensively to understand innate immune responses [9,10,11,12]. Insects are also important models in ecological research due to their sheer abundance in terrestrial ecosystems.

Here I call for increased synthetic research uniting two major fields of insect evolutionary ecology, the relatively new and rapidly growing field of ecological immunology, and the long-established field of plant-herbivore interactions. Much research attention has been and continues to be focused on the effects of host plant traits on herbivore-parasite interactions; however, relatively few authors have directly measured immune response itself, and published research so far has been restricted to larval Lepidoptera. A mechanistic approach to studying multitrophic interactions that incorporates measurement of the immune response is important. Interactions between plants and herbivorous insects are among the dominant ecological interactions in many terrestrial ecosystems. These lower trophic level interactions have dramatic effects on higher trophic interactions, in particular herbivore immune response to pathogen infection and endoparasitism. Host responses to pathogens and endoparasites influence population dynamics, colonization, and persistence, thus playing important roles in the structure and function of communities [13,14,15,16]. Basic research in plant-herbivore-parasite interactions is also important for applications in agroecosystems, in which herbivorous insects along with other invertebrates are major causes of economic damage and parasites function as biological control agents [17,18,19]. 

## 2. Evolution of Herbivore Diet Breadth and the Potential Role of Parasites and Pathogens

At least 90% of known herbivorous insect species are restricted to feeding on plant species belonging to three or fewer families [20]. Such species are often referred to as specialists, and specialist insect species may be further referred to as monophagous (feed successfully on a single host species or genus) or oligophagous (feed successfully on a “few” host species, genera, or families). Conversely, herbivore species that can feed successfully on plants from several families are referred to as generalists, or polyphagous. Referring to an insect species as a specialist or generalist can be problematic for multiple seasons, including the lack of complete information regarding host plant range of many species and errant host records for presumed specialist species. For example, populations of *Manduca sexta *(Lepidoptera: Sphingidae), a presumed Solanaceae specialist, have been recorded on *Proboscidea *(Martyniacaeae), belonging to a plant family that is classified in a different order [21]. Moreover, individuals of a species considered to be polyphagous may specialize on only a single host plant species [22,23,24]. Although flexibility exists in defining a species as a specialist or generalist, herbivore species that develop on a narrow range of host plant species with similar chemical and nutritional traits are primarily considered specialists, while generalist species have the ability to consume plants expressing a broad range chemical and nutritional traits [20,25].

The evolution of narrowed diet breadth in insect herbivores ranks among the most studied, and most varied, areas of research in evolutionary ecology [26], due in part to research supporting two competing groups of models derived from opposing viewpoints of the relative strength of selection provided by lower [27] and higher [28] trophic levels. “Bottom-up” models posit that coevolution between the nutritional and defensive traits of plants and the behavioral and physiological traits of herbivores may potentially explain narrowed feeding ranges [27,29,30]. In particular, adaptation to specific plant secondary metabolites by a herbivore population or species can reduce competition among herbivores and increase metabolic efficiencies. Specialist herbivores that have adapted to process or avoid specific metabolites outperform generalist herbivores when consuming diets containing the metabolites [20,31,32,33,34,35,36,37], at the expense of being restricted to those diets. Moreover, numerous comparative studies examining both generalist and specialist herbivore-natural enemy complexes have found that generalist complexes respond more poorly to plant metabolites than specialist complexes [38,39,40,41], generally due to the herbivore’s reduced nutritional quality to the natural enemy.

“Top-down” models hypothesize that the mortality caused by natural enemies is an important selective force of all insects, and is potentially a driving selective force in herbivore host range as well [28,42,43]. In particular, specialist natural enemies such as many pathogens and endoparasitic arthropods (endoparasitoids) may exert considerable pressure on herbivore host range as the primary sources of herbivore mortality in many ecosystems [44]. Evidence that pathogens and endoparasitoids may contribute to both narrowed and broadened host ranges comes from examination of the effect of parasitism risk and status on herbivore host selection behavior and how that behavior affects subsequent interactions with endoparasites [45,46]. The parasite-altered behavior of generalist herbivores has provided many insights in this regard. For instance, *Grammia incorrupta *(Lepidoptera: Arctiidae) larvae parasitized by tachinid endoparasitoids in early instars were less attracted to feeding on diets containing toxic plant compounds (hypothesized to reduce immune response) and more attracted to plant species containing antioxidants (hypothesized to improve immune response, see below) [47]. However, *G. incorrupta* larvae parasitized in later instars increased consumption of the same toxic compounds, suggesting a self-medication behavior [47,48]. From an evolutionary and ecological standpoint, it is likely that host selection in natural systems by individual herbivores of several species can be influenced by parasitism risk and status, with individuals making decisions that reduce parasite success more likely to survive and reproduce [43,49,50] even if forced to develop on low-quality host plants [51]. Over time, these decisions may have increased the chances of insect species becoming specialist herbivores, feeding primarily on plant species that provide metabolites that directly or indirectly reduce susceptibility to natural enemies.

## 3. Assays for Measuring Immune Responses in Herbivorous Insects

The insect immune response fits into two categories: (i) the humoral response, in which circulating antimicrobial peptides, RNAs, and lyzosomes are upregulated in response to the presence of viral, bacterial, and fungal pathogens [7,52,53]; and (ii) the cellular response, in which circulating hemocytes phagocytose microbial pathogens and envelop larger foreign bodies [8,54,55]. Both responses rely on recognition of an invasive body, and the upregulation of genes involved in immune response. 

Several assays can be used to measure humoral and cellular responses; careful and accurate measurement of the innate immune response of generalist and specialist insect herbivores is necessary to draw conclusions related to the effects of plant traits. The appropriate assay used to measure an herbivore’s immune response depends on several factors, including the herbivore’s morphology, physiology, and developmental stage, and the objectives of the research (*i.e.*, examining susceptibility to endoparasitoids or viruses) The use of multiple different assays can demonstrate tradeoffs between different immune system traits responding to the same challenges, or how immune response traits appropriate to different challenges (e.g., a virus or an endoparasitic arthropod) each may vary due to individual plant traits (*sensu *[56]). 

The effect of plant traits on the immune response of herbivores has been tested primarily using three techniques (with few published exceptions [56,57], only the cellular responses of larval Lepidoptera to endoparasitoids have been tested for these purposes), which are described in Section 3.1 and Section 3.2. While each can be used to measure immune response in both parasitized or unparasitized herbivores, the innate immune response is best tested in parasite-free animals. These techniques have proven to be very practical in studies that have shown correlations between these assays and field parasitism [58] and susceptibility to endoparasites [59]. Nonetheless, laboratory assays of immune response should be carefully interpreted as some studies have reported no correlation between immune assays and field parasitism success [60,61].

### 3.1. The Prophenol Oxidase Pathway

One of the primary pathways in the both the humoral and cellular immune responses is the prophenol-oxidase (PPO) cascade, which results in the oxidation of tyrosine derivatives such as L-DOPA by activated phenol oxidase (PO), and deposition of melanin on the invasive body [12,62,63]. The product of the PPO pathway is measured using absorbance in spectrophotometric assays. Hemolymph samples are incubated in buffer solution with serine proteases such as chymotrypsin [64] added to activate PO. A substrate such as L-DOPA is added to allow the enzymatic oxidation to proceed. The change in absorbance over time in the product is measured using spectrophotometry, and correlated with enzyme activity and thus immune response. The measurement of PO activity is strongly associated with susceptibility to certain pathogens and arthropod endoparasitoids [59,65], but is not correlated with susceptibility to other pathogens (such as viruses [57,66]). Measurement of PO activity is less parasite-specific than the two following assays, both of which simulate endoparasitoids. Assays of PO activity assays require only hemolymph samples rather than destroying the insect to find and remove an implant, which provides four advantages to the remaining implant-based techniques: (1) it is more easily performed in natural systems; (2) there is no wait for melanization in the hemocoel, and the sample isn’t lost if the insect dies during implantation; (3) one insect can be tested multiple times; (4) a parasitized insect can be tested, then kept alive to allow continued parasite development or emergence.

### 3.2. Simulation of Solitary Endoparasitoids

Solitary endoparasitoids are those in which single offspring emerge successfully from a single host; the successful endoparasitoid offspring may emerge alone because it was the sole invader, or it killed competing larvae inside its host [67,68]. Solitary development is considered the ancestral trait in Hymenopteran endoparasitoids [69], which more commonly exhibit this lifestyle compared to other endoparasitoid orders [68]. 

Solitary endoparasitoids have been simulated using 2.0 × 0.20 mm nylon microfilament implants placed in the hemocoel of the target insects for a pre-determined amount of time (as short as 1 hr [65,70]) during which encapsulation and melanization of the implant both occur. Implants are photographed upon removal, and the size of the hemocyte capsule and the color of the melanized implant can both be measured using various imaging software packages such as ImageJ, Image Pro, and Adobe Photoshop. Color is scored using grey color caused by melanization, and a non-implanted control microfilament is used as a comparison for both color and size after encapsulation and melanization.

### 3.3. Simulation of Gregarious Endoparasitoids

Gregarious development, the successful development of at least two endoparasitoid larvae in a single host, is considered a derived character in the Hymenoptera [69] and a general feature of endoparasitoids belonging to other orders such as Coleoptera and Diptera [68]. Gregarious development may be the result of superparasitism (several adult female parasitoids insert eggs into the same individual host, or a single female repeatedly oviposits into the same individual host) or large clutches of eggs inserted during one oviposition event.

**Figure 1:**
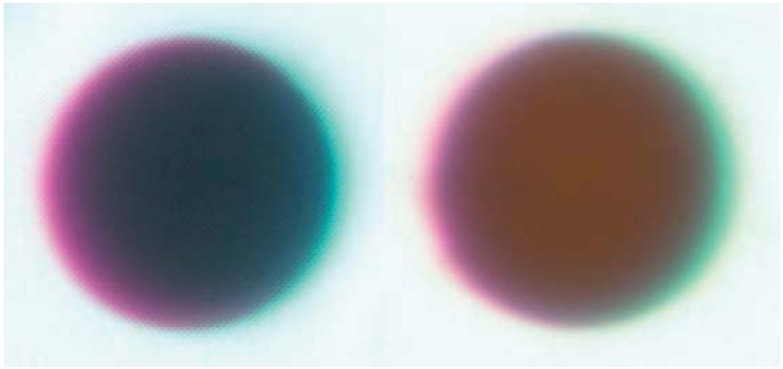
Representative beads dissected from the generalist *Spodoptera eridania* (Lepidoptera: Noctuidae) larvae reared upon (**left**) *Taraxacum officinale*, a high-quality diet, and (**right**) *Plantago lanceolata*, a low-quality diet [75]. Host plants were determined to be high or low quality based on observations of growth rate, and defensive chemistry of the two plant species. Photos by E.C. Lampert.

Gregarious endoparasitoids have been simulated by injecting a group of glass beads in physiological saline into the hemocoel. Sephadex chromatography beads, such as A-25 beads with a diameter of 40–120 µm, are commonly used [71,72,73]. A fixed volume of 10–15 µL saline + beads is injected into the hemocoel, and beads are individually recovered from caterpillars dissected after a pre-determined amount of time has passed for melanization to occur. As with filament implants, the size and color of the capsule can both be measured using images of each bead. Beads are often dyed red to facilitate retrieval; in this case the red color of melanized and control beads is compared to calculate melanization (Figure 1). 

## 4. Host Plant Traits and the Herbivore Immune Response

Plants provide the major nutritional compounds required for herbivorous insect growth and maintenance, such as carbohydrates, lipids and amino acids. In addition, plants produce a variety of secondary metabolites not used in primary metabolism that have important ecological effects on both herbivores and their interactions with each other and occupants of higher trophic levels [20,27,74]. Plant quality traits include a combination of major nutritional compounds and secondary metabolites, as well as morphological and architectural traits.

The identity and quantities of plant secondary compounds consumed by herbivores have been shown repeatedly to influence their interactions with both predatory and parasitic/pathogenic natural enemies (reviewed in [76,77,78]). These bottom-up tritrophic interactions can be broadly defined as direct, in which exposure to a plant compound within the herbivore has a positive or negative effect on the natural enemy, or indirect, in which the herbivore’s size or quality has been influenced by the compounds. Direct deleterious effects of plant compound sequestration and metabolism on parasitoids and pathogens have been demonstrated repeatedly and reviewed by other authors [77]. Here I focus on indirect effects of plant primary and secondary compounds on potential parasites and pathogens as mediated by enhanced and compromised innate immune responses of herbivores. Species discussed in examples in Section 4.1, Section 4.2, Section 4.3 and Section 4.4 are all larval Lepidoptera, as appropriate published research to date has been exclusive to this taxon.

### 4.1. Plant Identity, Plant Quality and Immune Responses of Generalist and Specialist Herbivores

Generalist and specialist herbivores both encounter variation in host plant quality traits while foraging. This variation may be the result of the variation provided by multiple plant species, and variation within a plant species, population, or individual. Generalist herbivores exhibit two foraging strategies; individuals of herbivore species considered generalists at the individual level may sample and consume many plant species [24,79], while those that are generalists at the population level may remain on a single plant individual or members of a single plant species [80,81]. Individual specialist herbivores generally follow a similar strategy to the latter, remaining on a single plant species or even a single plant individual throughout their lifespan [20]. 

Foraging on several plants species can provide herbivores with wide qualitative variation in nutritional and defensive compounds. A mixed diet throughout an individual’s lifespan provides many benefits from a multitrophic perspective, such as improving fecundity, and growth rate [82,83], and pharmacophagy (see [48], Section 2). However, a mixed diet may not necessary improve immune response. For instance, generalist *Parasemia plantaginis* (Lepidoptera: Arctiidae) larvae fed a 3-species mixed diet had a weaker immune response than those feeding on single plant species [84]. In this case, the mixed diets may have diluted the intake of compounds beneficial to growth and the immune response.

Plant defensive and nutritive traits can vary dramatically at the population level and the individual plant level (through ontogenetic changes and herbivore-induced changes as well as variation among tissues). This variability exposes herbivores consuming a single plant species to a wide variety of biotic conditions, even under similar environmental conditions. *Epirrita autumnata *(Lepidoptera: Geometridae) a generalist that feeds on several deciduous tree species [85], with cyclic population outbreaks, has provided several insights in this regard. Over several seasons and outbreaks, *E. autumnata *immune response has been found to be higher on high-quality trees [61], low-quality trees [65,86], induced trees [70], and alternative tree species [87]. The generally enhanced immune response on low-quality diets in this species (with the exception of [61]) may reflect a tradeoff between growth and immune response, as larvae stressed by diet quality may allocate higher proportions of resources to survival. 

Plant quality is often correlated with immune responses of both specialists and generalists. A survey of published research examining 10 generalist and specialist Lepidoptera species found that cellular immune response (measured as PO activity, implants, and bead injections (Table 1)) was higher in 5 species (counting *E. autumnata *[61]) when reared on or collected from a “high quality” or preferred plant, as determined by measuring other fitness correlates such as growth rate and larval or pupal mass. Only the generalist *E. autumnata *showed a reduced response and the others showed no change in immune response when reared on high-quality plants compared to low-quality plants (Table 1). The general association between high-quality diets and strong immune responses in this limited sample broadly fits a pattern showing that invertebrates experiencing favorable conditions also show strengthened immune responses [88,89]. Plant identity was associated with significantly strengthened or weakened immune responses in all of the specialist species and all of the generalist species except *Grammia incorrupta *(which showed a consistent immune response regardless of host plant species) (Table 1), supporting generally the hypothesis that lower trophic levels influence higher trophic interactions.

**Table 1:** Effects of host plant species and host plant quality on immune response of 9 specialist and generalist Lepidopteran herbivores.

Herbivore Species	Diet Breadth	Plants Used	Measurement	Immune Response	Citation
*Epirrita autumnata*	generalist	*Betula pubescens *and alternate tree hosts	implants	varied among cultivars and species, higher in induced and low-quality trees	[65,70,87]
				higher in high-quality trees	[61]
		high and low quality *Betula pubescens*	PO activity	no effect	[65]
*Eupoecilia ambiguella*	specialist	*Vitis vinifera *cultivars	PPO activity	varied among cultivars	[56]
*Grammia incorrupta*	generalist	three forb species	beads	no difference among plants	[73]
*Junonia coenia*	specialist	*Plantago lanceolata*,* P. major*	beads	higher when fed *P. major*	[90]
*Manduca sexta*	specialist	*Nicotiana tabacum *and *Proboscidea louisianica*	beads	higher on *N. tabacum*	[91]
*Orgyia antiqua*	specialist	two *Salix *spp.	implants	no effect	[92]
*Parasemia plantaginis*	generalist	four forb species	implants	higher on *Lactuga *and *Rumex*	[84]
*Pieris rapae*	specialist	wild and cultivated *Brassica*	egg encapsulation	reduced on induced plants, highest on Brussels sprouts	[93]
*Plutella xylostella*	specialist	four *Brassica *cultivars	PO activity	varied among cultivars, no relation to quality	[94]
*Trichoplusia ni*	generalist	*Brassica oleracea* and *Cucumis sativa*	PO activity, hemocyte #	no effect on PO, higher hemocytes on *B. oleracea*	[66]

### 4.2. Plant Nutritional Resources and Antioxidants May Enhance Melanization

Melanization and encapsulation responses of insects presented with foreign implants have been clearly linked to nutritional status. Starvation has been shown to reduce immune responses [4,57,94], as have low-quality diets that induce poor nutritional efficiencies [73,90,95]. Nutritional macromolecules such as amino acids and proteins provided by plants have important effects on herbivore growth and fitness [96], the role of these molecules in enhancing immune responses has received recent attention as well. The quality and quantity of nutritional resources are both important to immune response; for example, high-quality protein may contribute more to the nitrogen pool that the immune response draws from [95].

The PPO cascade that leads to melanization of foreign objects generates abundant reactive oxygen species that can harm the insect [97,98,99]. Plant-provided antioxidant compounds such as flavonoids, phenolics, and carotenoids remove these reactive oxygen species and have been shown to enhance immune response [100,101]. The effects of antioxidants on the immune response are of particular interest in evolution of host plant choices of generalist species. For instance, encapsulation ability of the generalist arctiid *Parasemia plantaginis *fed 3 plant species and an artificial diet was highest when fed plant species contributing compounds high in antioxidant activity such as flavonoids and carotenoids [84]. Moreover, encapsulation responses of other generalist caterpillars reared on *Taraxacum officinale *and *Malva parviflora *have been shown to be consistently high [73,75], and both plants are known to produce high levels of antioxidant flavonoids [102,103]. As mentioned above (Section 2), *Grammia incorrupta *larvae parasitized by dipteran endoparasitoids preferentially select flavonoid-rich *M. parviflora *over plants providing defensive compounds that can be sequestered [73].

Further evidence that plant antioxidants and other secondary compounds increase encapsulation and melanization comes from comparisons between herbivores reared on whole plants and artificial diets; recipes for the latter include primary nutrients, preservatives, and antibiotics but lack plant secondary compounds unless added separately. Both generalist and specialist herbivores benefit from diets enriched with these compounds. Weakened immune responses have been found in both generalist [73,84] and specialist herbivores [90] reared on artificial diets compared to plant diets. Thus, although a possible link between antioxidants and a strengthened immune response has been observed in a limited number of studies, further research is needed to determine the extent to which parasitism status induces a preference for plants high in antioxidants compared to other compounds.

### 4.3. Plant Defensive Chemistry and Herbivore Immune Response

One of the key differences between specialist and generalist herbivores is response to consuming plant defensive compounds [20]. Many specialist herbivores possess enzyme systems highly specialized to metabolize specific substrates. Substrate-specialized enzyme systems may be ineffective at metabolizing compounds structurally dissimilar to the substrate, causing specialists to respond poorly to novel compounds. Generalist herbivores are able to consume many more plant species and their various associated compounds due to broad-substrate enzyme systems [104,105]. Although substrate-specific enzymes are present in generalist herbivores [106], the prevalence in generalists of broad-substrate enzyme systems often makes them more susceptible to deleterious effects of defensive compounds compared to specialists. The reduced performance of specialists consuming novel compounds, and both specialists and generalists consuming large amounts of defensive compounds can include a weakened immune response.

Metabolizing large amounts of consumed plant compounds may be energetically expensive regardless of enzyme system specificity. These costs may divert needed resources away from immune responses. Increased dietary levels of glucosinolates and iridoid glycosides, two plant compounds associated with reduced digestive efficiencies [73,90,107,108] have been shown to have detrimental effects on the immune response of several generalist and specialist Lepidopteran species (Table 2). 

Performance measures such as growth rate, size, nutritional indices were also reduced along with immune response as higher doses of these compounds were consumed [75,90,93]. One specialist, *Ceratomia undulosa *(Lepidoptera: Sphingidae), was fed leaf discs supplemented with a novel compound (catalpol), with toxic effects of this compounds potentially contributing to its weakened immune response at a low dose [75]. A negative correlation between hydrolyzable tannins in host tree leaves and *Epirrita autumnata *immune response (with a positive correlation between hydrolyzable tannins and growth) was the only exception found to the general pattern of immune response mirroring other performance measures as a function of host plant chemistry [86], likely due to a strong tradeoff in this species between investments in growth *versus *immune response. 

Physiological and behavioral differences between generalists and specialists may explain instances in which the immune response of the former is not influenced by secondary compound consumption. Immune response of the grazing generalist *Grammia incorrupta* is not affected when consuming high doses of either iridoid glycosides or pyrrolizidine alkaloids (references in Table 2). Unlike many other generalists and most specialists, individual *G. incorrupta* sample several plants providing potentially several distinct chemical classes. The consistently high immune responses of *G. incorrupta* measured regardless of diet may be explained by broad-substrate enzyme systems that do not compete for resources with the immune response, unique enzyme systems in the immune response itself, or some other unique physiological trait of this species. More research is needed to determine if other generalists with a similar feeding style (e.g., other Arctiidae and several groups of Orthoptera) are similarly able to maintain a high immune response regardless of type and dose of dietary defensive compounds.

**Table 2:** Effects of plant secondary metabolites on immune response of specialist and generalist Lepidopteran herbivores.

Herbivore species	Diet Breadth	Plant Compound	Immune Response	Citation
*Parasemia plantaginis*	generalist	antioxidants	strengthened with increasing amounts consumed	[84]
*Epirrita autumnata*	generalist	flavonoids	no effect	[86]
		hydrolyzable tannins	weakened with increasing amounts consumed	[86]
*Pieris rapae*	specialist	glucosinolates	weakened in induced plants	[93]
*Junonia coenia*	specialist	iridoid glycosides	negatively correlated with amount consumed and sequestered	[90]
*Melitaea cinxia*	specialist	iridoid glycosides	positively correlated with amount consumed	[109]
*Grammia incorrupta*	generalist	iridoid glycosides	no effect	[73]
*Ceratomia catalpae*	specialist	iridoid glycosides	negatively correlated with amount sequestered	[75]
*Ceratomia undulosa*	specialist	iridoid glycosides	weakened with increasing amount consumed	[75]
*Grammia incorrupta*	generalist	pyrrolizidine alkaloids	no effect	[47]

### 4.4. Tradeoffs between Defensive Compound Sequestration and Herbivore Immune Response

Sequestration of plant secondary compounds, storing them in the hemocoel or other tissues in concentrations higher than the plant producing them, is common among both specialist and generalist herbivores [110,111], although specialist herbivores may sequester higher concentrations of plant compounds compared to generalists [43,111,112]. Sequestration is noted as an effective form of chemical defense against predators, pathogens, and endoparasitoids [111]. However, several cases have been found in which endoparasitoids are as or more successful in hosts sequestering higher levels of plant compounds compared to those in hosts that do not sequester [113,114,115]. Smilanich *et al*. [90] have proposed the “vulnerable host hypothesis” to explain this phenomenon, positing that defensive compound sequestration and the immune response can be antagonistic metabolic processes. 

**Figure 2:**
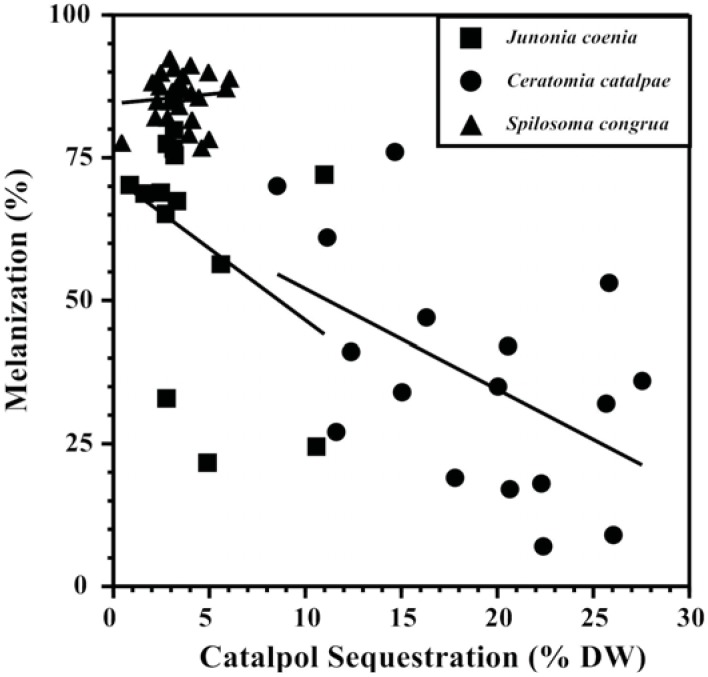
Tradeoffs between defensive chemistry and immune response of three Lepidopteran herbivores support the “vulnerable host hypothesis” [90]. Catalpol sequestration is negatively correlated with melanization ability of the specialists *Junonia coenia *(Nymphalidae) and *Ceratomia catalpae *(Sphingidae), but not the generalist *Spilosoma congrua* (Arctiidae).

Competing metabolic demands between sequestration and immune responses that show a tradeoff between the two processes are central to supporting the “vulnerable host hypothesis.” Plant compounds sequestered in the hemocoel are often modified by enzymes before storage (e.g., [116]) and cross gut epithelia with the help of transport proteins to reach their destination, both energetically expensive processes that may compete with immune response as well as a variety of other life functions [117,118,119]. Evidence that sequestration is energetically expensive was provided by path analysis models revealing that the specialist *Junonia coenia *(Lepidoptera: Nymphalidae) experienced reduced respiration rates as the amount of the iridoid glycoside catalpol sequestered increased [90]. Increasing amounts and concentrations of catalpol sequestered by *J. coenia *has been associated repeatedly with reduced nutritional efficiency and weakened immune response ([75,90,108], Figure 2,), while simultaneously reducing susceptibility to invertebrate predators [120,121]. The use of catalpol sequestration to test the “vulnerable host hypothesis” has been extended to two other herbivores as well; melanization response was negatively associated with sequestration in the specialist *Ceratomia catalpae *(Lepidoptera: Sphingidae),but not the generalist *Spilosoma congrua *(Lepidoptera: Arctiidae) (Figure 2). Specialized *C. catalpae* enzyme and carrier systems may require more energy for accumulating high catalpol concentrations (5x the concentrations accumulated by *S. congrua*), while *S. congrua *may lack these energy-intensive transport systems. 

## 5. Future Directions

The integration of ecological immunity and bottom-up effects of plant traits on herbivore-natural enemy interactions is relatively young (most research in this area has been published within the last decade) and open to expansion in several directions. Publications to date that directly measure herbivore immune response in regards to plant traits have focused almost exclusively on larval Lepidoptera, with floral traits and honeybee immune response as one exception [122]. Although larval Lepidoptera are particularly well-studied from both the perspectives of diet breadth evolution and immune responses, broader themes can be revealed by investigating other insect orders with particularly well-studied herbivorous species of economic and ecological importance (e.g., the Hymenoptera, Diptera, Hemiptera, Orthoptera, and Coleoptera). *Drosophila melanogaster *(Diptera: Drosophilidae) has been studied extensively as a model testing effects of starvation and diet resources on immune response, and yeast strain (presumably nutritional quality) has been shown to affect immune response [123].

Field tests in natural populations, particularly well-resolved natural systems (e.g., *Depressaria pastinacella* and *Utetheisa ornatrix* and their respective host plants, reviewed in [124,125], will further reveal the selective forces of plant traits on herbivore immune responses. With the exception of the *Betula pubescens*-*E. autumnata* system and a neotropical Lepidoptera assemblage [58], research on the effects of plant species and genotype on innate immune response itself have been primarily confined to laboratory settings. One reason for the lack of field studies is that the study of natural populations prevents experimental control of plant traits, requiring correlative relationships between plant traits and immune responses. Nonetheless, the study of natural populations can allow the assessment of correlation between the measured immune response and population-level parasitism success; a strong relationship has been found in one study [58] whereas no relationship has been found in another [61]. A second reason is that natural systems are not convenient for placing or recovering implants or beads. Hemolymph samples for PPO measurement are ideal in these settings.

Biological control by pathogens (including nematodes, viruses, bacteria, and fungi) and endoparasitoids and breeding/transgenic plants that express anti-herbivore traits are both important components of integrated pest management programs [17]. Plant traits such as defensive compounds that are detrimental to natural enemies lead to a potential incompatibility between these pest management strategies, and detrimental effects on natural enemies have been shown in several studies (reviewed in [76,78,126]). Careful examination of immune response can elucidate whether detrimental interactions between biocontrol agents and pest-resistant plants are direct or indirect, and potentially make these strategies more compatible. Plant traits have been linked to immune response in important field and greenhouse pests, such as the specialist *Manduca sexta *[127], and the generalist *Trichoplusia ni *[66], although not in agroecosystems. Potential candidates for further study include generalist Lepidoptera (e.g., *Heliothis virescens*, *Helicoverpa zea*, and *Spodoptera *spp., all Noctuidae), Orthoptera, and Hemiptera (e.g., Aphididae) which attack several crop species that are well-studied in plant-herbivore interaction ecological research.

## 6. Conclusions

Host plant traits are important biotic factors with strong influences on the ecology and evolution of herbivores, and their specialist natural enemies such as pathogens and endoparasitoids. Generalist and specialist herbivores can respond very differently to host plant defensive and nutritive traits, and these varied responses may be apparent in higher trophic level interactions as well. A detailed understanding of the effect of plant traits on herbivore immune responses provides a mechanistic explanation for the oft-observed tritrophic interactions among plant, herbivores, and endoparasitic natural enemies, and can produce important insights into the role of biotic and abiotic influences on disease susceptibility and transmission. 
